# Coronary artery bypass surgery with stent removal and coronary endarterectomy using a saphenous vein graft as an adventitial substitute (‘flooring’) for reconstruction of the left anterior descending artery: a case report

**DOI:** 10.1093/ehjcr/ytag496

**Published:** 2026-07-09

**Authors:** Mayu Yamamoto, Takahide Yoshio, Kenji Wada, Tomoya Uchimuro, Shunichiro Takanashi

**Affiliations:** Department of Cardiac Surgery, Kawasaki Saiwai Hospital, 31-27 Omiya-cho, Saiwai-ku, Kawasaki, Kanagawa 212-0014, Japan; Department of Cardiac Surgery, Kawasaki Saiwai Hospital, 31-27 Omiya-cho, Saiwai-ku, Kawasaki, Kanagawa 212-0014, Japan; Department of Cardiovascular Surgery, JR Tokyo General Hospital, 2-1 Yoyogi, Shibuya-ku, Tokyo 151-8528, Japan; Department of Cardiac Surgery, Kawasaki Saiwai Hospital, 31-27 Omiya-cho, Saiwai-ku, Kawasaki, Kanagawa 212-0014, Japan; Department of Cardiac Surgery, Kawasaki Saiwai Hospital, 31-27 Omiya-cho, Saiwai-ku, Kawasaki, Kanagawa 212-0014, Japan

**Keywords:** Case report, Coronary endarterectomy, In-stent restenosis, Adventitial loss, Coronary artery bypass grafting, Stent removal

## Abstract

**Background:**

Coronary endarterectomy with stent removal followed by arterial reconstruction is a surgical option for complex in-stent restenosis (ISR). However, this strategy generally assumes preservation of the coronary adventitia, and management of adventitial loss during stent removal has rarely been described.

**Case summary:**

A 67-year-old man on haemodialysis presented with exertional angina 1 year after percutaneous coronary intervention to the left anterior descending artery (LAD). Coronary angiography demonstrated severe ISR of the LAD with complex multivessel disease, and urgent off-pump coronary artery bypass grafting was performed. During coronary endarterectomy with stent removal, dense adhesion between the stent and vessel wall caused unavoidable loss of the coronary adventitia over a 10 mm segment. A longitudinally opened saphenous vein graft (SVG) was used as an adventitial substitute (‘flooring’) to reconstruct the LAD, followed by bypass using an *in situ* left internal thoracic artery with an onlay patch technique. The postoperative course was uneventful, and follow-up angiography confirmed excellent graft patency. The patient remained asymptomatic at 3-month follow-up.

**Discussion:**

This case highlights a salvage technique for unexpected adventitial loss during coronary stent removal. The use of a SVG as an adventitial substitute—the ‘flooring’ concept—combined with arterial onlay reconstruction may represent a feasible option in selected patients with complex ISR.

Learning points

- Coronary endarterectomy with stent removal may result in loss of the coronary adventitia, a rare but clinically relevant complication.
- When adventitial loss occurs, a saphenous vein graft may serve as a biological substitute to restore structural continuity of the coronary vessel wall.

## Introduction

With increasing complexity of percutaneous coronary intervention, a growing number of patients present with in-stent restenosis (ISR) unsuitable for repeat catheter-based revascularization. In such cases, coronary artery bypass grafting (CABG) combined with coronary endarterectomy and stent removal has been established as an effective surgical option.^1–3^ This technique generally assumes preservation of the coronary adventitia, which provides structural integrity and facilitates safe arterial reconstruction. However, separation of a chronically implanted stent from the vessel wall can be unpredictable, particularly in the presence of advanced fibrosis. To date, there is limited guidance on how to manage situations in which the adventitia is lost during stent removal.

Although onlay patch reconstruction is commonly used after coronary endarterectomy, these techniques generally assume preservation of the native adventitia.

We report a rare case in which adventitial loss of the left anterior descending artery (LAD) occurred intraoperatively and was successfully managed using a saphenous vein graft (SVG) as an adventitial substitute. Based on this experience, we propose the concept of coronary vessel wall ‘flooring’, in which a SVG is used to restore structural continuity after adventitial loss.

## Summary figure

**Figure:**
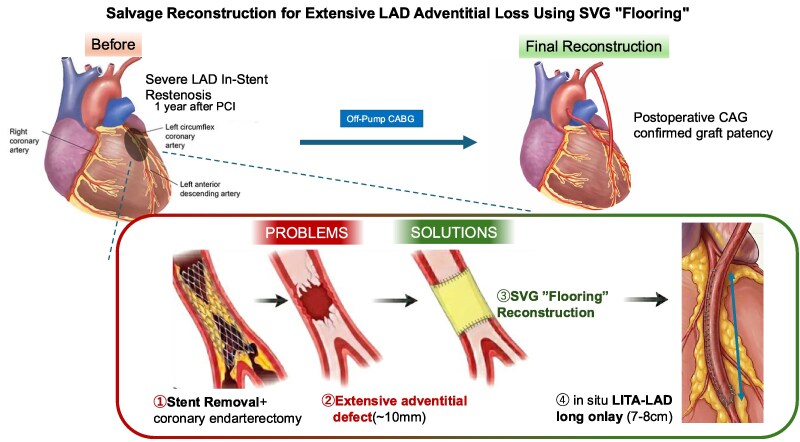


## Case presentation

A 67-year-old man receiving intermittent haemodialysis presented with progressive exertional chest pain. On physical examination, the patient was haemodynamically stable with no signs of heart failure; no cardiac murmurs or peripheral oedema were noted. One year earlier, he had undergone percutaneous coronary intervention with drug-eluting stent implantation in the LAD. On admission, electrocardiography showed ST-segment depression in the anterior leads. Coronary angiography demonstrated severe ISR of the LAD and critical 90% stenosis of the left main trunk. The right coronary artery was hypoplastic, and the left circumflex artery—which supplied the corresponding territory—had 90% stenosis at two separate sites. In addition, a 90% lesion was present in the diagonal branch. The anatomical complexity was high, with a SYNTAX score of 30.

After multidisciplinary Heart Team discussion, surgical revascularization with CABG was deemed the most appropriate strategy, and off-pump CABG was performed via median sternotomy.

The LAD had an internal diameter of ∼2.5 mm, and the intimal quality was moderately diseased. Following stent removal, coronary endarterectomy was performed over an ∼5-cm segment and extended proximally and distally until areas with relatively preserved intimal quality were reached to obtain a stable luminal surface.

At the site of stent extraction, dense adhesion between the stent and the vessel wall resulted in unavoidable loss of the adventitial layer. The adventitial defect was limited to ∼10 mm in the mid-portion of the endarterectomized segment, while the remaining portions of the reconstructed LAD preserved the native adventitia.

**Figure 1:**
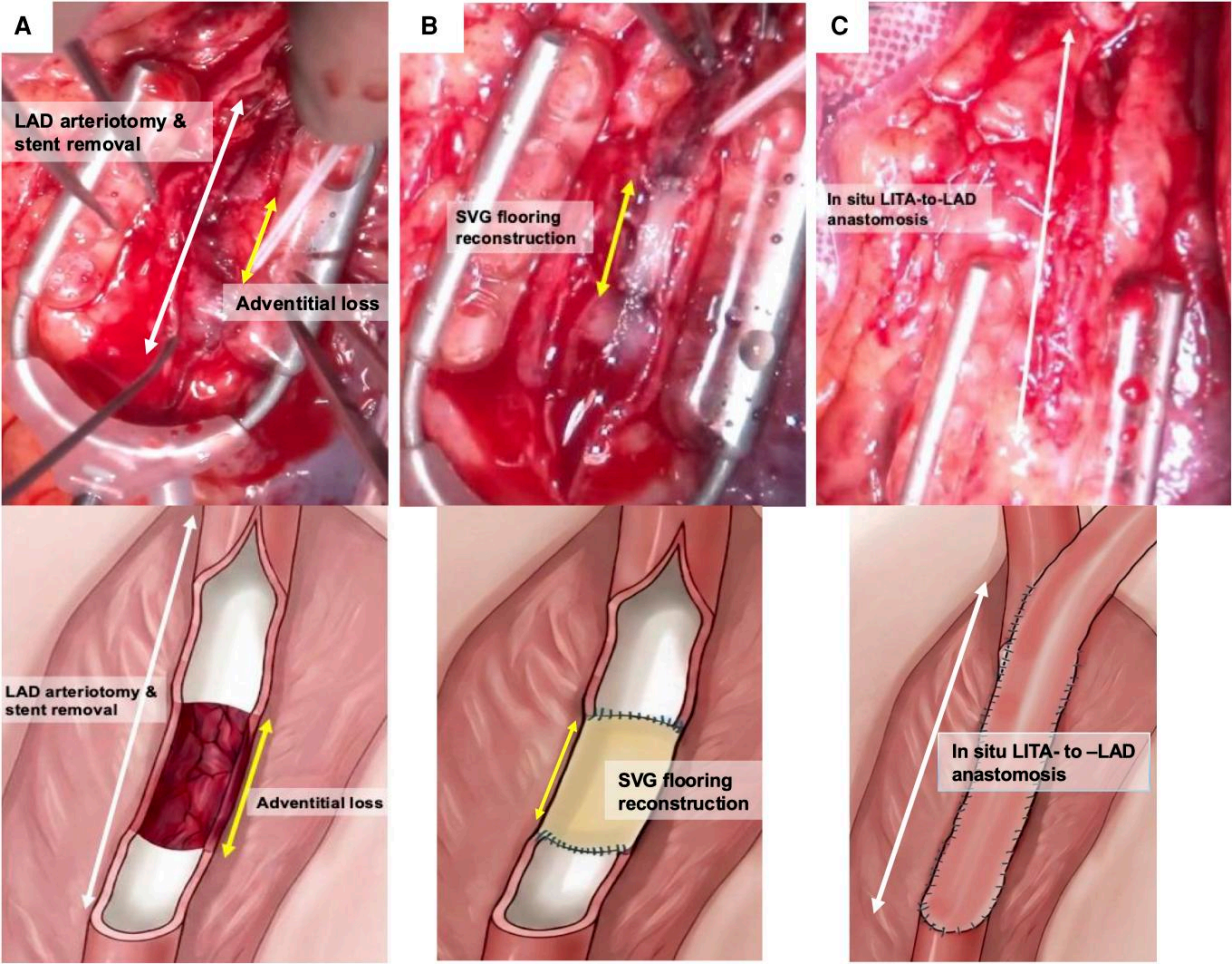
Intraoperative reconstruction of the left anterior descending artery after stent removal and coronary endarterectomy. (*A*) Intraoperative view of the left anterior descending artery following longitudinal arteriotomy, 5-cm coronary endarterectomy, and removal of the previously implanted stent. The stent was densely adherent to the vessel wall, and its extraction resulted in complete loss of the left anterior descending artery adventitia. A 10-mm longitudinal segment of complete adventitial defect is visible along the 2.5-mm-diameter left anterior descending artery, leaving the arterial wall markedly fragile. (*B*) Application of the ‘flooring’ technique. A tailored saphenous vein graft was positioned over the adventitial defect. The saphenous vein graft was continuously sutured to the residual adventitia on both sides of the left anterior descending artery using 8-0 Prolene, thereby restoring external structural support along the 10-mm defect. (*C*) Final reconstructed configuration. After restoration of adventitia continuity with the saphenous vein graft flooring, an *in situ* left internal thoracic artery graft was anastomosed to the reconstructed left anterior descending artery segment using an onlay patch technique, achieving complete revascularization. LAD, left anterior descending artery; SVG, saphenous vein graft; LITA, left internal thoracic artery.

To restore structural continuity, a longitudinally opened SVG was continuously sutured with 8-0 polypropylene to the preserved adventitia on both sides of the defect to completely cover the adventitial loss, thereby creating a reinforced vessel wall configuration. This reconstruction, in which the SVG functions as an adventitial substitute, is referred to by the authors as ‘flooring’. The intraoperative findings and the ‘flooring’ reconstruction technique are illustrated in *Figure 1*. Subsequently, the left internal thoracic artery was used as an *in situ* graft and anastomosed to the reconstructed LAD using an onlay patch technique. Intraoperative transit-time flow measurement demonstrated a mean graft flow of 22 mL/min, a pulsatility index of 1.8, and a diastolic filling of 79%, indicating satisfactory graft function.

**Figure 2:**
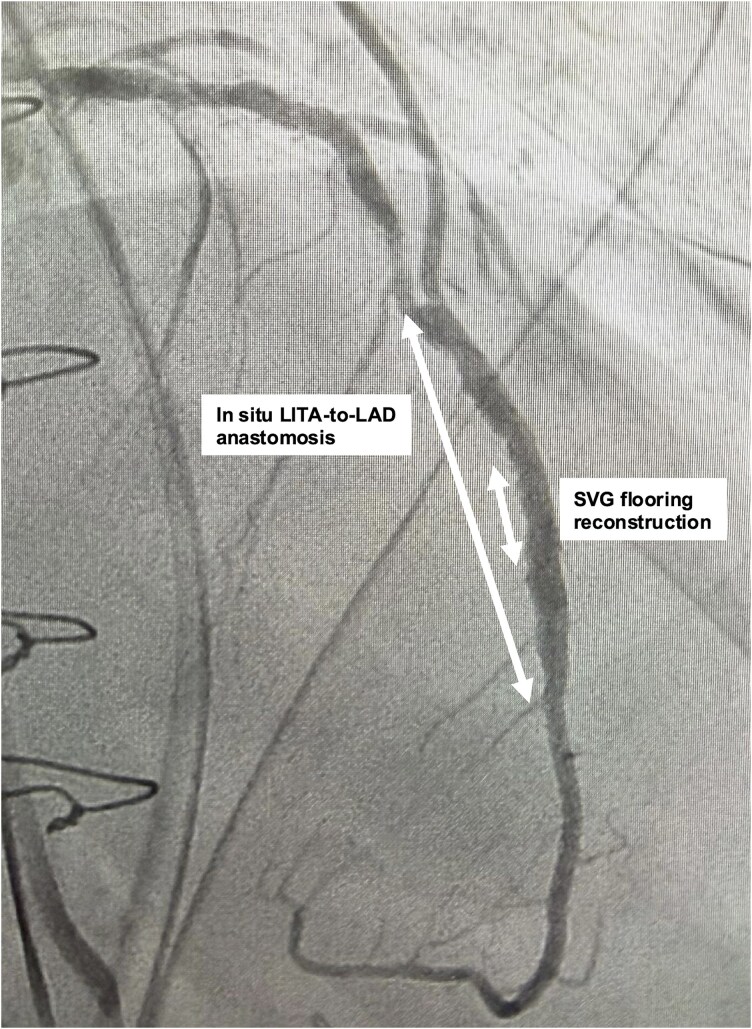
Postoperative coronary angiography. Coronary angiography performed on postoperative Day 4 demonstrating a widely patent *in situ* left anterior descending artery-to-left anterior descending artery graft with satisfactory distal runoff and no evidence of anastomotic stenosis or aneurysmal change. LAD, left anterior descending artery; LITA, left internal thoracic artery.

The postoperative course was uneventful. Anticoagulation with heparin was initiated on postoperative Day 1. From postoperative Day 2, antithrombotic therapy was switched to aspirin and warfarin. Coronary angiography performed on postoperative Day 4 demonstrated excellent graft patency without anastomotic narrowing, confirming successful reconstruction of the LAD (*Figure 2*).

## Discussion

This report describes a practical surgical solution for an uncommon but potentially serious complication of coronary stent removal—loss of the coronary adventitia.

Coronary endarterectomy has re-emerged as an important adjunct to CABG for diffusely diseased coronary arteries and complex ISR^1–3^; however, the procedure remains technically demanding and may be associated with vessel wall injury. While vessel injury is a recognized complication, most reports focus on luminal reconstruction and do not describe how to manage cases in which the structural integrity of the vessel wall itself is compromised.

In standard endarterectomy procedures, plaque removal is performed along the plane between the diseased intima and the adventitia, and preservation of the adventitial layer is generally assumed.^1^ However, in the present case, dense adhesion between the implanted stent and the vessel wall precluded identification of a clear dissection plane, resulting in unavoidable loss of the adventitial layer. This finding suggests that, in selected cases of severe ISR, even meticulous surgical technique may not prevent adventitial disruption.

Beyond its mechanical role, the adventitia is increasingly recognized as an active regulator of vascular injury responses, including inflammatory and thrombotic processes.^4^ Disruption or loss of the adventitial layer may alter the local haemostatic environment by exposing prothrombotic substrates and extracellular matrix components to circulating blood, potentially increasing the risk of platelet activation and thrombus formation.^4,5^ Although direct clinical evidence linking adventitial loss to thrombotic complications in coronary surgery is limited, this biological framework provides a plausible mechanistic explanation for adverse outcomes following extensive vessel wall injury.

Based on these considerations, reconstruction of the defect using some form of biological tissue was considered necessary. Potential reconstructive materials included the internal thoracic artery, SVG, and autologous pericardium. Although the internal thoracic artery may theoretically provide suitable vascular tissue for reconstruction, preservation of the conduit for coronary bypass grafting was prioritized in this case.

A SVG was considered suitable because it could be harvested relatively easily and retained the structural characteristics of a vascular conduit, making it appropriate for patch-like reconstruction of the coronary wall defect.^6,7^ Autologous pericardium is also used in patch reconstruction; however, concerns regarding calcific degeneration and the lack of native vascular architecture made it less favourable than SVG in the present setting.

Particular attention was paid to graft orientation and handling to minimize thrombogenicity and ensure structural stability. The graft was longitudinally opened and positioned with the intimal surface facing the luminal side. It was then sutured to the residual adventitia so as to completely cover the adventitial defect, thereby providing external structural support while preventing luminal collapse. We refer to this reconstruction concept as ‘flooring’, in which the SVG serves as a structural substitute for the lost adventitia, restoring external coronary wall continuity.

Potential long-term concerns include aneurysmal degeneration, thrombus formation, patch remodelling, and incomplete endothelialization of the reconstructed segment.^5^ Careful postoperative antithrombotic management may therefore be important, although the optimal regimen remains to be established.

This report is limited by its single-case nature and the relatively short follow-up period of 3 months, which precludes definitive assessment of long-term durability. Further experience and longer-term follow-up are required to validate the safety and durability of this technique.

## Conclusion

When coronary adventitial loss occurs during stent removal and endarterectomy, reconstruction using a SVG as an adventitial substitute (‘flooring’) may provide structural support and allow safe arterial revascularization. This approach offers a potential salvage option for selected patients with complex ISR.

## Patient’s perspective

The patient reported marked improvement in exercise tolerance after surgery and expressed satisfaction with the treatment outcome. He appreciated the detailed explanation of the surgical procedure and agreed to publication of this case.

## Data Availability

The data underlying this article are available from the corresponding author upon reasonable request.

## Lead author biography

Dr Mayu Yamamoto is a cardiovascular surgery resident in Japan. Her clinical interests include CABG, complex coronary reconstruction, and valvular surgery. She is particularly interested in surgical strategies for complex coronary artery disease, including ISR and coronary endarterectomy.

## Notes

**Consent:** The authors confirm that written consent for submission and publication of this case report including images and associated text has been obtained from the patient in line with the COPE guidelines.
